# Improving prognostic evaluations in patients with stage IIIb light chain cardiac amyloidosis: role of haemodynamic parameters

**DOI:** 10.1186/s13023-024-03451-z

**Published:** 2025-01-13

**Authors:** Jingyi Li, Yang Lu, Xiqi Xu, Zhuang Tian, Jian Li, Shuyang Zhang

**Affiliations:** 1 Department of Cardiology, State Key Laboratory of Complex Severe and Rare Diseases, Peking Union Medical College Hospital, Chinese Academy of Medical Sciences and Peking Union Medical College, No. 1 Shuaifuyuan, Dongcheng District, Beijing, 100730 China; 2 Department of Hematology, Peking Union Medical College Hospital, Chinese Academy of Medical Sciences and Peking Union Medical College, No. 1 Shuaifuyuan, Dongcheng District, Beijing, 100730 China

**Keywords:** Light chain cardiac amyloidosis, Heart failure, Haemodynamic parameters, Survival analysis, Echocardiography

## Abstract

**Background:**

There is no unified prognostic scoring system for light chain cardiac amyloidosis (AL-CA), particularly stage IIIb AL-CA. This study aimed to use invasive haemodynamic information to investigate markers that can more accurately evaluate the prognosis of patients with stage IIIb AL-CA.

**Methods:**

In this retrospective cohort study, we conducted invasive haemodynamic measurements concurrently with myocardial biopsies to diagnose AL-CA. We used Cox regression analysis and time-dependent receiver operating characteristic curve analysis to study the associations between these measurements and overall mortality. Echocardiographic parameters were also recorded and analysed via logistic regression to explore their relationships with haemodynamic changes.

**Results:**

Although traditional haemodynamic parameters, such as the cardiac index (CI), pulmonary artery wedge pressure (PAWP), pulmonary artery pressure, and vascular resistance, did not correlate with mortality, the PAWP/CI ratio emerged as a vital prognostic marker. Patients with a PAWP/CI ratio above 11 mmHg/L/min/m^2^ had markedly poorer survival. Kaplan‒Meier analysis highlighted the prognostic significance of the ratio, revealing distinct survival differences. Furthermore, logistic regression confirmed that echocardiographically measured pulmonary artery systolic pressure independently correlated with increases in the PAWP/CI ratio.

**Conclusions:**

In stage IIIb AL-CA patients, the PAWP/CI ratio, which surpasses traditional haemodynamic indicators, significantly predicts all-cause mortality, emphasizing its prognostic value. Our findings suggest that echocardiography-derived PASP could alternatively reflect the PAWP/CI ratio.

**Supplementary Information:**

The online version contains supplementary material available at 10.1186/s13023-024-03451-z.

## Introduction

Light-chain cardiac amyloidosis (AL-CA) is a rare but severe form of amyloidosis characterized by the deposition of immunoglobulin light chains in the heart, leading to restrictive cardiomyopathy and heart failure [1]. Cardiac involvement occurs in approximately 70% of newly diagnosed AL amyloidosis patients, often presenting as heart failure with preserved ejection fraction (HFpEF). Typical clinical signs include exertional dyspnoea, arrhythmias such as atrial fibrillation, and conduction abnormalities [2]. Research has shown that the prevalence of this condition increases among older populations, typically manifesting around the median age of 76, with a marginally higher occurrence in males. Reports indicate a consistent annual incidence rate for AL of 1.2 cases per 100,000 individuals [3]. Before the advent of daratumumab, the median overall survival (OS) of patients with cardiac stage III AL-CA was 7 months, with OS rates of 73%, 55%, 46%, and 29% at 3, 6, 12, and 24 months, respectively [4]. Administering daratumumab markedly enhances the survival outcomes of individuals with AL-CA [5]. Currently, the assessment of AL-CA patient prognosis relies primarily on the 2004 and 2012 Mayo staging systems [6, 7]. The Mayo 2004 staging system is primarily based on cTn and NT-proBNP levels. Previous studies have shown that approximately 19% of patients are classified as having stage IIIb disease, with a median survival of only 4.6 months. The Mayo 2012 staging system introduced the difference in serum free light chains (dFLCs) as an additional criterion, classifying approximately 23% of cases as stage IV, with a median survival of 5.8 months. The disease is primarily diagnosed by a combination of clinical presentations, imaging, and histological confirmation through myocardial biopsy. With technological advancements, cardiovascular magnetic resonance imaging and strain echocardiography are progressively showing their distinct benefits in evaluating structural and functional alterations in the heart, monitoring disease progression, and guiding treatment [8, 9]. However, there is currently no universally accepted scoring system to assess the prognosis of stage IIIb patients. From a clinical perspective, haemodynamic measurements may offer greater value in this regard. There is no consensus about which specific haemodynamic parameters are significantly prognostic. The current research included patients with stage IIIb AL-CA and collected haemodynamic information, aiming to outline the haemodynamic properties of patients with stage IIIb AL-CA and identify haemodynamic predictors that better assess prognosis.

## Materials and methods

### Study population

We retrospectively included patients diagnosed with AL-CA stage IIIb at Peking Union Medical College Hospital from May 2020 to March 2023. All the patients underwent comprehensive evaluations by experienced haematologists and cardiologists, who determined the necessity of a myocardial biopsy to confirm the diagnosis of AL-CA. During the myocardial biopsy procedure, haemodynamic measurements were also conducted. According to the 2004 Mayo Cardiac Staging System [6, 10], a diagnosis of stage IIIb disease was made when cTnI was greater than or equal to 0.1 μg/L and NT-proBNP was greater than or equal to 8500 ng/L. The exclusion criteria were as follows: (1) had concurrent malignant tumours; (2) had multiple myeloma, Waldenström's macroglobulinemia, or other lymphoplasmacytic proliferative disorders; (3) had extensive myocardial infarction; (4) had other cardiomyopathies; (5) had congenital heart disease; and (6) had moderate or severe valvular stenosis.

### Baseline clinical characteristics and outcomes

This evaluation included an assessment of patient demographics, comorbid conditions, medical history, laboratory results, treatments, and echocardiography. The criteria for determining the involvement of organs other than the heart are provided in the Supplementary Methods. The eGFR was calculated with the CKD-EPI formula to estimate the glomerular filtration rate. Recent recommendations in echocardiography were followed for measurements [11], resulting in the collection of data on maximal thickness of the left ventricular wall (MLVWT), diameter of the left ventricle at end-diastole (LVEDD), percentage of blood pumped out of the left ventricle during each contraction (LVEF), velocity of tricuspid regurgitation (TRV), and Doppler imaging of the mitral annulus tissue. Further calculations for pulmonary artery systolic pressure (PASP) and the left ventricular mass index (LVMI) were performed, with specific formulas provided in the Supplementary methods. In the current analysis, the focus was on evaluating all-cause mortality as the outcome.

### Haemodynamic measurements and definitions

Baseline haemodynamic data were acquired via right heart catheterization (RHC). All the measurements, including right atrial pressure (RAP), right ventricular systolic pressure, right ventricular end-diastolic pressure, systolic pulmonary arterial pressure (sPAP), diastolic pulmonary arterial pressure (dPAP), mean pulmonary arterial pressure (mPAP), and pulmonary artery wedge pressure (PAWP), were obtained with patients in a supine position, at rest, and at the end of expiration without holding their breath. Using a continuous cardiac output (CO) monitoring module, CO was determined by averaging three consecutive measurements for precision. Additionally, both blood pressure and heart rate were documented. Subsequent calculations included the cardiac index (CI), stroke volume, pulmonary vascular resistance (PVR), systemic vascular resistance (SVR), and diastolic pressure gradient (DPG), and the specific formulas that were used are provided in the Supplementary methods. On the basis of the latest guidelines and relevant literature [12, 13], we defined haemodynamic parameters as abnormal according to the following criteria: CI < 2.2 L/min/m^2^, PAWP > 15 mmHg, RAP > 8 mmHg, mPAP > 20 mmHg, and PVR > 2 Wood units.

### Statistical analyses

R (version 4.3.1) and RStudio were utilized for conducting statistical analyses. Categorical information is displayed in terms of frequencies and percentages, whereas continuous variables following a normal distribution are presented as means and standard deviations. Nonnormally distributed variables are summarized as medians and interquartile ranges. Patient prognosis was assessed via Cox regression, which calculates hazard ratios (HRs) along with 95% confidence intervals (CIs), and the assumption of proportional hazards was verified via the 'survival' package. Variables were selected for univariate Cox analysis on the basis of clinical experience and the literature. Multicollinearity was addressed by running separate multivariate Cox regression models. Time‒dependent receiver operating characteristic (ROC) curve analysis revealed the best cut-off values for haemodynamic variables related to all-cause mortality via the Youden index. The log-rank test was used to compare the haemodynamic groups. Logistic regression was used to analyse echocardiographic parameters, providing odds ratios (ORs) and 95% CIs. Statistical significance was indicated by *P* values less than 0.05.

## Results

### Baseline demographic and clinical characteristics

Table 1 summarizes the characteristics of the 28 patients in the study; the average age was 59 years, and the patients were predominantly male (n = 20). Patients had similar physiques, with an average BMI of 23.35 kg/m^2^ and a BSA of 1.83 m^2^. Most patients (79%) were diagnosed with λ-type AL-CA, with 46% having multiple organ involvement. A median dFLC of 385.0 mg/L was also associated with a high disease burden. Most patients were classified into NYHA functional class III-IV (79%), with a median NT-proBNP level of 14,703 ng/L. Echocardiography revealed that 68% of patients had a normal LVEF, whereas 25% had an LVEF less than 40%. The average E/e' ratio was 22, and the average PASP was 37 mmHg. Structural alterations included left ventricular wall thickening, with an average MLVWT of 15.5 mm and an LVMI of 154 g/m^2^. The left ventricular dimensions were mostly normal. The comorbidities included arrhythmias (32%, mainly atrial fibrillation and ventricular tachycardia), hypertension (n = 8), diabetes (n = 5), coronary artery disease (n = 6), and stroke (n = 3). Thirty-nine percent of patients had an estimated glomerular filtration rate (eGFR) lower than 60 ml/min/1.73 m^2^. It took an average of 346 days from the appearance of symptoms to receive a diagnosis. Treatment included daratumumab-based regimens for 21 patients and bortezomib-based regimens for 7 patients.

**Table 1:** Baseline characteristics of patients with light-chain cardiac amyloidosis of mayo stage IIIb

Variables	Overall population (n = 28)
Demographics	
Age, years	59 ± 10
Male/female, n	20/8
BMI, kg/m^2^	23.35 ± 3.66
BSA, m^2^	1.83 ± 0.18
Clinical	
NYHA III-IV, n (%)	22 (79)
Arrhythmia, n (%)	9 (32)
HTN/DM/CAD/stroke, n	8/5/6/3
eGFR < 60 ml/min/1.73 m^2^	11 (39)
λ type, n (%)	22 (79)
Involved organs ≥ 2, n (%)	13 (46)
Interval of onset to diagnosis, day	346 ± 262
Daratumumab/bortezomib^*^, n	21/7
Laboratory	
NT-ProBNP, ng/L	14703 (11081, 30596)
cTnI, μg/L	0.358 (0.171, 0.683)
dFLC, mg/L	385.0 (177.6, 604.1)
Echocardiography	
LVEF, n (%)	
≥ 50%	19 (68)
41–49%	2 (7)
≤ 40%	7 (25)
E/e'	22 ± 7
PASP, mmHg	37 ± 11
LVEDD, mm	42 (40, 45)
MLVWT, mm	15.5 ± 2.5
LVMI, g/m^2^	154 ± 39
Hemodynamics	
Heart rate, bpm	85 ± 12
Systolic BP, mmHg	109 ± 18
Diastolic BP, mmHg	72 ± 12
CI, L/min/m^2^	1.9 ± 0.5
Stroke volume, ml/min	43 ± 15
PAWP, mmHg	21 ± 8
RAP, mmHg	12 ± 6
RV systolic pressure, mmHg	45 ± 15
RV end-diastolic pressure, mmHg	13 ± 6
sPAP, mmHg	44 ± 15
dPAP, mmHg	22 ± 8
mPAP, mmHg	30 ± 10
PVR, Wood units	2.4 (1.8, 3.4)
SVR, Wood units	20.4 (17.1, 23.6)
DPG, mmHg	1 (−1, 4)

Categorical variables were expressed as counts and percentages. For normally distributed data, we showed continuous variables by their mean and standard deviation. When data were not normally distributed, we reported these variables by their median and interquartile range [median (IQR)]. BMI indicates body mass index; BP, blood pressure; BSA, body surface area; CAD, coronary artery disease; CI, cardiac index; cTnI, cardiac troponin I; dFLC, difference between involved and uninvolved serum free light chains; DM, diabetes mellitus; DPG, diastolic pressure gradient; dPAP, diastolic pulmonary artery pressure; E/e’, the ratio of transmitral E to mitral annular e’ velocities; eGFR, estimated glomerular filtration rate; HTN, hypertension; IQR, interquartile range; LVEDD, left ventricular end-diastolic diameter; LVEF, left ventricular ejection fraction; LVMI, left ventricular mass index; MLVWT, maximal left ventricular wall thickness; mPAP, mean pulmonary artery pressure; NT-proBNP, N-terminal pro-B-type natriuretic peptide; NYHA, New York Heart Association; PAWP, pulmonary artery wedge pressure; PASP, pulmonary artery systolic pressure; PVR, pulmonary vascular resistance; RAP, right atrial pressure; RV, right ventricular; sPAP, systolic pulmonary artery pressure; SVR, systemic vascular resistance.

^*^Daratumumab means patients received a first-line treatment regimen based on daratumumab; bortezomib means patients opted for a bortezomib-based first-line treatment.

### Initial haemodynamic properties

**Fig. 1:**
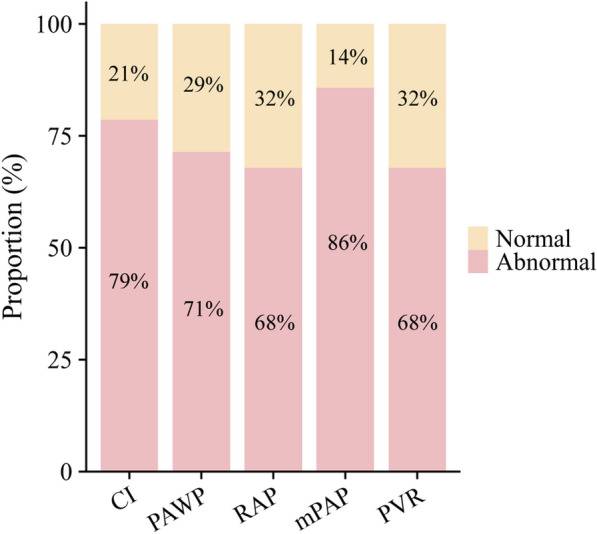
The distribution of various abnormal hemodynamic parameters throughout the entire cohort. Criteria for abnormal hemodynamic parameters were established as follows: CI < 2.2 L/min/m^2^, PAWP > 15 mmHg, RAP > 8 mmHg, mPAP > 20 mmHg, and PVR > 2 Wood units. CI indicates cardiac index; mPAP, mean pulmonary artery pressure; PAWP, pulmonary artery wedge pressure; PVR, pulmonary vascular resistance; RAP, right atrial pressure.

Table 1 shows the initial haemodynamic properties of the patients. Heart rate and blood pressure were generally normal, with no significant tachycardia or hypotension. The average CI was slightly low (1.9 L/min/m^2^), whereas the PAWP and RAP were elevated (21 mmHg and 12 mmHg, respectively). These findings suggest mild impairment of systolic function but significant diastolic dysfunction due to amyloid deposition. As shown in Fig. 1, elevated mPAP was observed in 86% of the patients, CI abnormalities in 79%, and PAWP in 71%. Both RAP and PVR abnormalities were present in 68% of the patients.

### Haemodynamic parameters and prognostic assessment

**Fig. 2:**
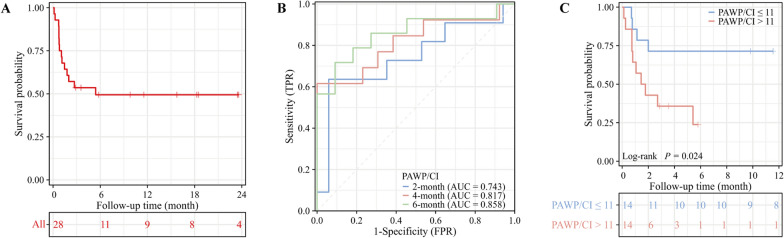
Kaplan–Meier Curve and Receiver Operating Characteristic Curve for Survival Analysis. **A** The Kaplan–Meier curve for overall survival in the whole study cohort. **B** Time-dependent receiver operating characteristic curve to evaluate PAWP/CI ratio performance at different time points. **C** The Kaplan–Meier curve stratified by the optimal cut-off value of PAWP/CI ratio obtained from the time-dependent receiver operating characteristic curve. AUC indicates area under the curve; CI, cardiac index; FPR, false positive rate; PAWP, pulmonary artery wedge pressure; TPR, true positive rate.

Figure 2A shows the survival status of the 28 patients over an average follow-up of 18.5 months (range 11.5–27.5 months). Fourteen patients (50%) died, with a median lifespan of 5.4 months; 11 died within 2 months. The other 14 patients had no adverse events. Univariate Cox analysis, which incorporates haemodynamic indices and additional prognostic covariates, revealed no significant correlations between NT-proBNP, cTnI, dFLC, eGFR, or treatment strategy and all-cause mortality, as shown in Table 2. Notably, the data in Table 2 also indicate that traditional haemodynamic parameters, including CI, PAWP, RAP, mPAP, and PVR, are not associated with all-cause mortality. Expanding upon this foundation, we introduced the PAWP/CI ratio as a novel parameter, which demonstrated a considerable association with the prognosis of patients (HR, 1.124 [95% CI, 1.035–1.221]; *P* = 0.005).

**Table 2:** Univariate cox proportional hazard analysis for all-cause mortality

Variables	Hazard ratio	95% confidence interval	*P* value
NT-proBNP	4.379	0.402–47.728	0.226
cTnI	1.259	0.481–3.298	0.639
dFLC	1.428	0.463–4.406	0.535
eGFR	0.340	0.062–1.878	0.216
Treatment bortezomib	Reference	Reference	–
Daratumumab	1.471	0.322–6.721	0.619
CI	0.332	0.096–1.148	0.082
PAWP	1.066	0.992–1.146	0.080
PAWP/CI ratio	1.124	1.035–1.221	0.005
RAP	1.076	0.986–1.174	0.099
mPAP	1.031	0.987–1.077	0.173
PVR	1.046	0.919–1.190	0.495

Based on clinical experience and prior researches, we strategically selected certain variables for univariate Cox regression analysis. CI indicates cardiac index; cTnI, cardiac troponin I; dFLC, difference between involved and uninvolved serum free light chains; eGFR, estimated glomerular filtration rate; mPAP, mean pulmonary artery pressure; NT-proBNP, N-terminal pro-B-type natriuretic peptide; PAWP, pulmonary artery wedge pressure; PVR, pulmonary vascular resistance; RAP, right atrial pressure.

Variables were subsequently chosen for multivariate Cox regression analysis on the basis of a *P* value of approximately 0.2. To avoid collinearity between the PAWP/CI ratio and the variables CI and PAWP, we designated NT-proBNP, eGFR, RAP, and mPAP as fixed variables. Building on this, we separately introduced the PAWP/CI ratio and the variables CI and PAWP to develop 2 distinct models, which were then analysed through multivariate Cox regression analysis, as shown in Table 3. In Model 1, none of the variables, including the CI or PAWP, were associated with all-cause mortality. Model 2 indicated a significant correlation solely between the PAWP/CI ratio and worse prognosis (HR, 1.276 [95% CI, 1.072–1.519]; *P* = 0.006).

**Table 3:** Multivariate cox proportional hazard analysis for all-cause mortality

Model 1			Model 2		
Variables	HR (95% CI)	*P*	Variables	HR (95% CI)	*P*
NT-proBNP	2.944 (0.103–84.192)	0.528	NT-proBNP	1.352 (0.043–42.587)	0.864
eGFR	0.604 (0.059–6.155)	0.670	eGFR	0.573 (0.060–5.465)	0.629
RAP	1.063 (0.897–1.260)	0.478	RAP	1.108 (0.936–1.313)	0.234
mPAP	0.950 (0.844–1.069)	0.392	mPAP	0.892 (0.777–1.025)	0.108
CI	0.360 (0.075–1.741)	0.204	PAWP/CI	1.276 (1.072–1.519)	0.006
PAWP	1.111 (0.981–1.258)	0.097	–	–	–

In the context of univariate analysis, variables with a *P* value around 0.2 were incorporated into the multivariate analysis. NT-proBNP, eGFR, RAP, and mPAP were fixed covariates. CI and PAWP was integrated into Model 1; PAWP/CI ratio was incorporated into Model 2. CI indicates Cardiac index; eGFR, estimated glomerular filtration rate; mPAP, mean pulmonary artery pressure; NT-proBNP, N-terminal pro-B-type natriuretic peptide; PAWP, pulmonary artery wedge pressure; RAP, right atrial pressure

### Relationship between the cut-off values of the PAWP/CI ratio and outcomes

Time-dependent ROC analysis was used to determine the most effective threshold value of the PAWP/CI ratio for prognosis prediction. Given that all outcomes in the cohort occurred within 6 months, we predicted the 2-month, 4-month and 6-month survival probabilities with time-dependent ROC analysis. Figure 2B reveals that the predictive capacity of the PAWP/CI ratio for 6-month survival rates was superior, with an area under the curve (AUC) of 0.858. The most advantageous threshold was determined to be 11 mmHg/L/min/m^2^, with a comprehensive presentation of these findings provided in Table S1. Patients were classified into distinct clusters on the basis of their PAWP/CI ratio, either being at or below 11 mmHg/L/min/m^2^ or exceeding it. As shown in Fig. 2C, the Kaplan‒Meier estimates revealed a statistically significant difference in survival probabilities (log-rank *P* = 0.024) between the two groups. Patients with a PAWP/CI ≤ 11 mmHg/L/min/m^2^ demonstrated superior survival outcomes compared with those with a ratio > 11 mmHg/L/min/m^2^.

### Echocardiographic parameters associated with elevated PAWP/CI ratios

We used whether the PAWP/CI ratio exceeded 11 mmHg/L/min/m^2^ as the outcome and performed logistic regression analysis to investigate echocardiographic parameters associated with elevated PAWP/CI ratios. As shown in Table 4, the univariate analysis indicated a substantial association of both LVEF (OR, 0.921 [95% CI, 0.846–0.984]; *P* = 0.027) and PASP (OR, 1.236 [95% CI, 1.102–1.462]; *P* = 0.002) with increased PAWP/CI ratios. The association of LVEF was not sustained in the multivariate model. Nonetheless, within the multivariate model, PASP remained a notable predictor (OR, 1.220 [95% CI, 1.082–1.447]; *P* = 0.005). E/e', the MLVWT, and the LVMI did not reach significance in the univariate analysis.

**Table 4:** Parameters of echocardiography associated with elevated pawp/ci ratios in the logistic regression model

Parameters	Univariate		Multivariate	
	OR (95% CI)	*P* value	OR (95% CI)	*P* value
LVEF	0.921 (0.846–0.984)	0.027	0.950 (0.846–1.051)	0.331
E/e'	1.066 (0.953–1.212)	0.285	–	–
PASP	1.236 (1.102–1.462)	0.002	1.220 (1.082–1.447)	0.005
MLVWT	1.330 (0.972–1.926)	0.094	–	–
LVMI	1.012 (0.992–1.034)	0.257	–	–

Guided by clinical judgment, we selected 5 echocardiographic parameters for logistic regression analysis. Subsequently, variables that showed statistically significant results were advanced to multivariate analysis. CI indicates cardiac index; E/e’, the ratio of transmitral E to mitral annular e’ velocities; LVEF, left ventricular ejection fraction; LVMI, left ventricular mass index; MLVWT, maximal left ventricular wall thickness; OR, odds ratio; PAWP, pulmonary artery wedge pressure; PASP, pulmonary artery systolic pressure; 95% CI, 95% confidence interval

## Discussion

AL-CA is a relatively rare disease that poses diagnostic challenges. Not all medical institutions are equipped to perform comprehensive haemodynamic assessments, making research on the haemodynamics of patients with AL-CA relatively rare. Our study specifically targeted patients with stage IIIb disease and revealed that their haemodynamics are characterized by a significant increase in biventricular filling pressure, whereas stroke volume and CI only slightly decrease.

The majority of patients included in our study were elderly or middle-aged males, which is consistent with the known demographic characteristics of this disease. Nearly all patients presented with significant heart failure symptoms. The markedly elevated levels of NT-proBNP and cTnI indicate severe cardiac injury in stage IIIb patients, with almost half of the patients also experiencing renal dysfunction, likely due to the combined effects of light-chain amyloidosis and impaired cardiac function. Additionally, the average time from symptom onset to diagnosis was nearly one year, suggesting a heavy disease burden for these patients and highlighting the ongoing challenges in diagnosing this condition. Echocardiography revealed that most patients still presented with an HFpEF pattern, with significantly impaired diastolic function and notably thickened ventricular walls, indicating that even in stage IIIb patients, a decrease in LVEF is relatively uncommon. However, haemodynamic data obtained through RHC revealed a significant reduction in CI for most patients, along with elevated PAWP, mPAP, and PVR, suggesting that both systolic and diastolic functions are notably compromised in stage IIIb patients.

Even though daratumumab has optimized the proportion and rate at which patients with AL-CA achieve a cardiac response, the early mortality rate among stage IIIb patients remains high at 22.8% [14]. In our cohort, all deaths were early deaths, whereas survivors responded exceptionally well to treatment without experiencing worsening heart failure or other adverse events. Therefore, identifying factors contributing to poor outcomes in stage IIIb patients can help clinicians identify those who fail to benefit from treatment early. Prior research has investigated how haemodynamic measurements can predict outcomes for patients suffering from AL-CA. The focus was on assessing whether these indicators could serve as reliable predictors for patient prognostics. A study published in 2013 involving 89 AL-CA patients identified the RAP as a primary predictor of free-heart transplantation survival [15]. In 2023, an extensive study including 198 individuals diagnosed with AL-CA demonstrated the substantial prognostic value of CI in forecasting outcomes such as all-cause mortality, the need for heart transplants, and the use of left ventricular assist devices more than other haemodynamic parameters. Although the PAWP is significantly elevated, it is not independently associated with prognosis [13]. In summary, the CI, which reflects systolic function, and the PAWP, RAP, mPAP, and PVR, which reflect diastolic function, are considered potentially associated with prognosis. However, the results from different cohort studies vary, likely due to the dynamic nature of patients' haemodynamic status, and a single baseline measurement may not provide enough information. This study focused on examining the role of haemodynamic data in patients with stage IIIb disease, as these patients are critically ill and require haemodynamic guidance in the intensive care unit. Additionally, we aimed to assess whether these data can be used to evaluate long-term prognosis in such patients. In our stage IIIb AL-CA cohort, we incorporated key haemodynamic parameters into the survival analysis, which yielded no meaningful results. We also discovered that the cardiac injury biomarkers NT-proBNP and cTnI were not associated with prognosis. Similarly, the eGFR, an indicator of renal function impairment, was not statistically significant according to the Cox regression model. Innovatively, we propose the PAWP/CI ratio as a new parameter. Compared with other conventional haemodynamic parameters, the PAWP/CI ratio better reflects the condition of patients whose PAWP is significantly elevated even when there is only a mild decrease in CI, thereby accentuating the extent of cardiac output impairment and diastolic dysfunction. The results of our investigation revealed an independent correlation between the newly identified PAWP/CI ratio and all-cause mortality among stage IIIb AL-CA patients. This study also identified a clear cut-off value for PAWP/CI, which demonstrated excellent predictive performance for 6-month all-cause mortality in AL-CA stage IIIb patients. Given the extremely short median survival and high early mortality rate in stage IIIb patients, this parameter and its cut-off value can be highly valuable in clinical practice. It can assist clinicians in more accurately assessing patient prognosis and adjusting treatment strategies in a timely manner.

Our research supports the implementation of haemodynamic measurements in all patients with stage IIIb AL-CA to better assess their prognostic outcomes. However, a significant number of medical institutions are unable to standardize the use of RHC, or some patients' financial situations do not support such procedures. Given that nearly all patients with cardiomyopathy undergo echocardiography and that the literature indicates that, in certain cases, echocardiography can partially substitute for RHC [16, 17], we further explored echocardiographic parameters that could replace the PAWP/CI ratio. Our findings indicate a direct association between elevated PASP and an increase in the PAWP/CI ratio, rather than LVEF or other indicators of cardiac structural abnormalities. PASP is calculated by experienced cardiologists via the measured tricuspid regurgitation velocity combined with an estimated RAP. Previous studies have shown a positive correlation between PASP measured by echocardiography and sPAP measured by RHC [18]. Additionally, the PASP has been found to predict the risk of sudden death in patients with pulmonary hypertension [18], suggesting that the PASP can, to some extent, serve as a noninvasive substitute for invasive haemodynamic measurements. These findings further suggest that diastolic dysfunction, not systolic dysfunction, leads to worse cardiac function and prognosis in patients with stage IIIb AL-CA. Although our results indicate that the MLVWT and LVMI are not linked to an increase in the PAWP/CI ratio, we believe that the ventricular wall abnormalities caused by amyloid deposition are related to haemodynamic abnormalities. We hypothesize that the lack of statistically significant results may be due to the inability of echocardiography to accurately measure cardiac amyloid deposition.

There are many clinical implications of our findings. First, the identification of a specific haemodynamic cut-off provides a tangible parameter for clinicians to target therapeutic interventions, potentially guiding treatment adjustments and monitoring strategies. Second, our research highlights the need for a fundamental change in handling stage IIIb AL-CA, suggesting a more haemodynamically informed approach that goes beyond conventional cardiac imaging and biomarker assessment. Finally, in the absence of haemodynamic results, echocardiography may serve as a potential alternative to RHC to some extent.

However, our study is not without limitations. The retrospective nature of the analysis, while comprehensive, is inherently subject to biases associated with historical data collection and patient selection. One of the other limitations is the restricted sample size, which inherently affects the generalizability of our findings. While we have endeavoured to ensure the robustness and validity of our analysis through rigorous statistical methods, the relatively small cohort may not fully represent the broader population. This limitation is particularly relevant when considering the heterogeneity of the conditions under investigation, where individual variations could significantly influence outcomes. Therefore, it is important to interpret our findings carefully, as broader and more varied samples could lead to different conclusions.

## Conclusions

The PAWP/CI ratio serves as a significant prognostic marker in patients with stage IIIb AL-CA, surpassing traditional haemodynamic parameters in predicting all-cause mortality. This finding highlights the importance of incorporating the PAWP/CI ratio into the prognostic assessment of stage IIIb AL-CA patients. Our results also indicate that the PASP measured by echocardiography may serve as an alternative to the PAWP/CI ratio.

## Supplementary Information

Supplementary Material 1.

## Data Availability

All data supporting the conclusions of this research article are included within the manuscript.

## Abbreviations

AL-CA: Light chain cardiac amyloidosis
dPAP: Diastolic pulmonary artery pressure
LVEDD: Left ventricular end-diastolic diameter
LVMI: Left ventricular mass index
MLVWT: Maximal left ventricular wall thickness
PASP: Pulmonary artery systolic pressure
sPAP: Systolic pulmonary artery pressure
TRV: Tricuspid regurgitation velocity
