# Supplementary material for: Improving prognostic evaluations in patients with stage IIIb light chain cardiac amyloidosis: role of haemodynamic parameters
Source: Orphanet J Rare Dis. 2025 Jan 13;20:19. doi: 10.1186/s13023-024-03451-z (PMC11727539; doi:10.1186/s13023-024-03451-z)
Supplement: Supplementary file 1 — Supplementary Material 1. [file 13023_2024_3451_MOESM1_ESM.docx]

**Supplemental methods**

**Criteria for organ involvement**

The criteria for determining the involvement of organs other than the heart were provided in Supplementary Methods: (1) renal involvement indicated by a 24-hour total urinary protein greater than 0.5 g, primarily consisting of albumin; (2) peripheral nerves characterized by symmetrical sensory and/or motor neuropathies in the lower limbs; and (3) autonomic nerves characterized by gastric emptying disorders, pseudointestinal obstruction, and dysregulation of excretion, unrelated to organ infiltration.

**Calculation of echocardiographic parameters**

Estimation of right atrial pressure (RAP): (1) when the right atrial size is normal and there is mild tricuspid regurgitation, the estimated RAP is approximately 5 mmHg; (2) when the right atrial size is mildly enlarged and there is moderate tricuspid regurgitation, the estimated RAP is approximately 10 mmHg; (3) when the right atrial size is significantly enlarged and there is severe tricuspid regurgitation, the estimated RAP is approximately 15 mmHg.

Pulmonary artery systolic pressure (PASP) is calculated using the following formula: PASP = 4 × tricuspid regurgitation velocity² + RAP

Left ventricular myocardial mass (LVM) and left ventricular myocardial mass index (LVMI) are calculated using the following formulas: LVM = 1.04 × [(left ventricular internal diameter + interventricular septal thickness + left ventricular posterior wall thickness)^3^ - left ventricular internal diameter^3^] × 0.8 + 0.6; LVMI = LVM / body surface area

**The formulas of calculating hemodynamic parameters**

Cardiac index = cardiac output / body surface area

Stroke volume = cardiac output ×1000 / heart rate

Pulmonary vascular resistance = (mean pulmonary artery pressure - pulmonary artery wedge pressure) / cardiac output

Systemic vascular resistance = (mean artery pressure - central venous pressure) / cardiac output

Diastolic pressure gradient = diastolic pulmonary artery pressure - pulmonary artery wedge pressure

**Table S1. Time dependent ROC Results of PAWP/CI ratio.**

| Time (month) | Cutoff (mmHg/L/min/m^2^) | Positive Predictive Value | Negative Predictive Value | Sensitivity | Specificity |
| --- | --- | --- | --- | --- | --- |
| 2 | 15 | 0.88 | 0.80 | 0.64 | 0.94 |
| 4 | 15 | 1 | 0.75 | 0.62 | 1 |
| 6 | **11** | 0.89 | 0.76 | 0.72 | 0.91 |
